# Workflow for Gene Overexpression and Phenotypic Characterisation in *Taraxacum kok-saghyz*

**DOI:** 10.3390/mps9010017

**Published:** 2026-01-24

**Authors:** Loredana Lopez, Michele Antonio Savoia, Loretta Daddiego, Paolo Facella, Elio Fantini, Linda Bianco, Simone Maci, Francesco Panara

**Affiliations:** 1 Trisaia Research Center, Italian National Agency for New Technologies Energy and Sustainable Economic Development, (ENEA), 75026 Rotondella, MT, Italy; loretta.daddiego@enea.it (L.D.); paolo.facella@enea.it (P.F.); elio.fantini@enea.it (E.F.); linda.bianco@enea.it (L.B.); 2 Department of Soil, Plant and Food Sciences, University of Bari Aldo Moro, Via Amendola 165/A, 70126 Bari, BA, Italy; michele.savoia@uniba.it (M.A.S.); s.maci1@phd.uniba.it (S.M.)

**Keywords:** *Taraxacum kok-saghyz*, genetic transformation, gene overexpression, natural rubber

## Abstract

*Taraxacum kok-saghyz* (Tks) is a promising plant species for natural rubber (NR) production and represents a model for studying NR biosynthesis in the *Asteraceae* family. The generation of transgenic plants overexpressing a gene of interest is a well-established strategy to investigate gene function and potential interactions. Here, we present a comprehensive workflow—from the construction of an overexpression vector to the generation, identification, and propagation of stable transgenic Tks lines. In addition, we describe a rapid and reliable method for quantifying NR content in transformed plants, providing essential phenotypic characterisation in this species.

## 1. Introduction

Natural rubber is a crucial industrial raw material, exhibiting distinctive mechanical properties, namely elasticity, resilience, and strength, that synthetic alternatives have yet to fully replicate. Its wide-ranging applications encompass medical devices, automotive components, construction materials, and specialised industrial products [1,2]. At present, over 90% of the world’s natural rubber originates from a single species, *Hevea brasiliensis*, which is cultivated almost exclusively in Southeast Asia [3]. This heavy reliance on a monoculture in a geographically limited area creates significant risks for global rubber supply. These risks include susceptibility to devastating diseases such as South American Leaf Blight (SALB), vulnerability to climate change, and socioeconomic instability affecting production and distribution chains [4].

In response to these challenges, the identification and development of alternative rubber-producing crops have become a strategic priority in both agricultural and industrial research. Among the most promising candidates is *Taraxacum kok-saghyz* (Tks), commonly known as the Russian dandelion. Native to Central Asia, Tks produces high-molecular-weight natural rubber (cis-1,4-polyisoprene) in its roots, which is chemically and functionally equivalent to rubber derived from *H. brasiliensis*. As a temperate species with a relatively short life cycle, Tks is well suited to cultivation in non-tropical climates, offering the potential for decentralised, climate-resilient, and regionally diversified rubber-production systems [5].

Despite its promising traits, including rubber content in its roots ranging from 5 to 20% dry weight and the co-production of valuable metabolites like inulin and secondary metabolites [6,7], Tks remains under-domesticated. Its commercial development is hindered by several agronomic, biological, and technical challenges. A key constraint is the limited availability of efficient and reproducible genetic transformation systems. Genetic engineering tools are essential not only for functional genomic studies but also for the accelerated breeding of Tks varieties with enhanced rubber yield, disease resistance, stress tolerance, and desirable agronomic characteristics [8].

To date, a number of transformation approaches have been explored in Tks, including *Agrobacterium tumefaciens*- and *Agrobacterium rhizogenes*-mediated methods [9,10]. Although hairy-root transformation offers rapid gene delivery and expression, it does not routinely enable the regeneration of whole plants [11]. Conversely, *A. tumefaciens* methods have demonstrated potential but frequently encounter challenges such as low transformation frequencies, genotype dependency, and inadequate regeneration efficiency [12]. Recent innovations, including the “cut–dip–budding” technique, have facilitated the streamlining of procedures and enhanced their applicability. As reported in [13], this method is a fast, tissue-culture-free technique that uses *Agrobacterium* to directly infect cut root segments. This generates transformed buds from hairy roots, making genetic modification simpler and quicker than with traditional methods. This technique bypasses lengthy steps such as callus induction by relying on the natural ability of Tks to form shoots from roots. However, challenges persist in the standardisation of protocols across genotypes.

In this study, we present a simple and optimised transformation protocol suitable for Tks. All the necessary steps—from the realization of the construct to the detailed description of the transformation and regeneration procedures, including explant type, bacterial strain, co-cultivation conditions, and selection regimes—are described. The protocol has been optimised for effectiveness across a range of genotypes including *Taraxacum brevicorniculatum*, a close Tks relative species [14], and is compatible with downstream molecular and phenotypic analyses. We included a step-by-step description of the protocol for the evaluation of rubber, inulin, and resin content. This work provides essential tools for the study of this emerging rubber crop and can be further adapted to applications such as genome editing, gene functional validation, and precision breeding.

Ultimately, this work describes a robust and reproducible workflow for generating transgenic Tks overexpression lines, supporting future functional analyses and applied research in this emerging rubber-producing species.

## 2. Experimental Design

**Figure 1:**
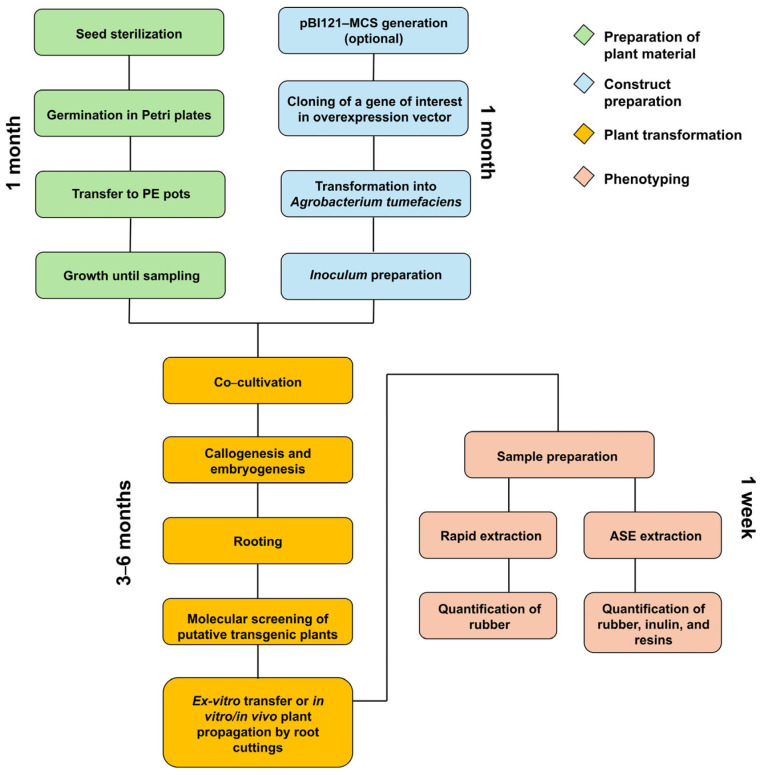
General workflow overview illustrating a scheme summarising the experimental design employed for Tks transformation.

A graphical summary of the whole procedure described in this work from gene overexpression to morphological characterisation in Tks is shown in Figure 1. The completion of an experiment can take from 4 to 12 months depending on the number of regenerated plants needed. The transformation of Tks is mediated by *A. tumefaciens* and transformed plants are obtained through somatic embryogenesis. The development and use of genetically modified plants are subject to specific regulatory frameworks that vary among countries. For the purposes of this study, it is assumed that any genetically modified plants obtained will be used exclusively for research purposes. All activities involving these plants are carried out in controlled and contained environments, in accordance with relevant biosafety regulations. Additionally, transformed plants can be clonally propagated as described here or can be backcrossed with wild-type plants in order to generate small populations for more accurate phenotyping. It has to be taken in consideration that Tks is generally self-incompatible [15,16,17,18]; thus, transformed plants cannot be backcrossed to the same wild-type plant used for transformation. This introduces a high degree of genetic variability that can be overcome with clonal propagation.

The transformation workflow described in this manuscript was successfully applied in [14]. Stable transgenic Tks and *Taraxacum brevicorniculatum* plants overexpressing the transcription factor TksMYC2 were generated. This enabled the functional characterisation of TksMYC2, demonstrating its regulatory role in specialised metabolism and natural rubber biosynthesis. Transgenic plants generated using this procedure showed significant alterations in the accumulation of sesquiterpene lactones, phenylpropanoids, and free fatty acids, and a marked increase in natural rubber content.

Furthermore, several experiments using this protocol were completed and will be published upon completion of phenotyping experiments.

Using the described procedure, we observed a callus induction rate ranging from 80 to 95% and a somatic embryo formation rate exceeding 75%. Over 50% of the obtained calli produced fully regenerated plantlets.

The overall transformation efficiency observed, defined as the proportion of explants that produce transgenic plants, was 35%. This result is due to the presence of some non-transformed calli that survive the antibiotic selection. Nevertheless, we do not suggest increasing the antibiotic dosage, as it may negatively affect even successfully transformed plants.

Among transformed plants, only 5–10% showed successful overexpression; that is to say, the expression of the gene of interest was at least two-fold higher in the transformed plants compared to the wild-type plants. Both the expression and phenotype of transformed plants were stable and consistent in our experiments. Transgenic plants were clonally propagated and clones exhibited stable transgene expression levels over time and among clones.

A difference in transgene expression level among different lines was observed, which is likely due to position effects. We did not observe gene-silencing effects in any of our regenerated lines.

These metrics quantitatively assess the performance of the workflow and can guide the experimental design in its future applications.

The main advantages when using the described procedures are as follows:

1. The whole procedure is fast, allowing the generation of new transformed plants in 3 to 6 months.
2. The in vitro culture procedure is simplified by reducing the number and complexity of necessary media.
3. The protocol is applicable to multiple *Taraxacum* species, increasing its versatility.
4. The use of somatic embryogenesis to regenerate transformed plants avoids the occurrence of chimerism as the somatic embryo derives from a single cell.

This procedure can be successfully applied for functional genomic studies in Tks, allowing for a better comprehension of genes and genetic networks involved in NR synthesis and understanding the possible strategies to improve rubber and other metabolites’ production in order to make Tks a suitable alternative species for NR production in temperate climates. Additionally, this procedure, with appropriate modifications, can be applied to other investigation strategies/approaches for functional genomics such as gene silencing or CRISPR-Cas9.

### 2.1. Materials

1. 1-Naphtalenacetic acid (NAA) (Merck, Darmstadt, Germany; Cat. no.: N0640).
2. Acetone (Merck, Darmstadt, Germany; Cat. no.:270725).
3. Agar (Merck, Darmstadt, Germany; Cat. no.: 5040).
4. Agarose (Merck, Darmstadt, Germany; Cat. no.: A9539).
5. *Agrobacterium tumefaciens* strain EHA105 (GoldBio, St. Louis, MO, USA; Cat. no. CC-225-5x50).
6. Aluminium foil weighing boats.
7. Augumentin (Amoxicillin/Clavulanic acid, 875 mg/125 mg- GSK, London, UK).
8. Bi-distilled sterile water.
9. Shrimp Alkaline Phosphatase, rSAP (New England Biolabs, Ipswich, MA, USA; Cat. no.: M0371).
10. Commercial bleach (5–10% NaClO).
11. Competent *Escherichia coli* cells, strain DH5alpha.
12. Absolute ethanol.
13. Gamborg B5 vitamin mixture (Duchefa, Haarlem, the Netherlands; Cat. no.: G0415).
14. Glycerol (Merck, Darmstadt, Germany; Cat. no.: G5516).
15. 0.75 M HCl.
16. 10 mN H_2_SO_4_.
17. Hexane (Merck, Darmstadt, Germany; Cat. no.: 1.04367).
18. Indole-3-acetic acid (IAA) (Merck, Darmstadt, Germany; Cat. no.: I2886).
19. InnuPrep Plant DNA Kit (Analytik Jena, IST Innuscreen GmbH, Berlin; Cat. no.: 845-KS-1060010).
20. InnuPrep Plant RNA Kit (Analytik Jena, IST Innuscreen GmbH, Berlin; Cat. no.: 845-KS-2060010).
21. Kanamycin (Merck, Darmstadt, Germany; Cat. no.: K1377).
22. LB Broth (Luria–Bertani Broth—Merk, Darmstadt, Germany; Cat. no.: 51208).
23. Liquid nitrogen.
24. MinElute PCR Purification Kit (Qiagen, Venlo, the Netherlands; Cat. no.: 28006).
25. Murashige and Skoog Basal Salt mixture (MS) (Merck, Darmstadt, Germany; Cat. no.: M5524).
26. pBI121 vector (TaKaRa Bio, Kusatsu, Japan).
27. 0.45 µm PTFE Whatman® filter (Cytiva, Marlborough, MA, USA; Cat. no.: 6784-2504).
28. Q5 High-Fidelity DNA Polymerase (New England Biolabs, Ipswich, MA, USA; Cat. no.: M0491L).
29. QIAwave Plasmid Miniprep Kit (Qiagen, the Netherlands; Cat. no.: 27204).
30. Rifampicin (Merck, Darmstadt, Germany; Cat. no.: R3501).
31. Size-exclusion resins (Sephadex G-50 or Sepharose CL-6B).
32. SuperScript™ IV Reverse Transcriptase (Thermo-Fischer Scientific, Waltham, MA, USA; Cat. no.: 18090010).
33. T4 DNA ligase (New England Biolabs, Ipswich, MA, USA; Cat. no.: M0202)
34. Trans-Zeatin (Duchefa, the Netherlands; Cat. no.: Z0917).
35. Tween-20 (Merk, Darmstadt, Germany; Cat. no.: P1379).
36. XbaI and SacI (New England Biolabs, Ipswich, MA, USA; Cat. no.: R0145L and R3156L).

### 2.2. Equipment

Standard laboratory equipment: analytical balance; autoclave; electroporator (MicroPulser Electroporator, model 1652100, Biorad, CA, USA); 4 °C, −20 °C, and −80 °C refrigerators/freezers; agarose gel electrophoresis apparatus; hot plate with magnetic stirrer; growth chambers; plant cultivation chambers; pipettes for micro-volumes; pipettors; laminar flow hood; chemical hood; orbital shaker; thermocycler; pH meter; refrigerated centrifuge; spectrophotometer (OD 600 nm); temperature-controlled incubators; water purification system; ultracentrifugal mill; ultrasonic cleaning bath; vibrating sieve; plastics and consumables for molecular biology and in vitro plant manipulation (i.e., transparent vessels for in vitro plant cultivation such as magenta boxes (Merck, Darmstadt, Germany; Cat. no.: C0542), Petri dishes, ~2 L polyethylene pots, spatulas, forceps, handles/blades, beakers, 250 mL Erlenmeyer’s flasks, and pipette tips).

ASE 200 accelerated solvent extractor (Dionex Corp., Now Thermo Fisher Scientific Inc., Waltham, MA, USA).

HPLC DX 300 system (Dionex, Sunnyvale, CA, USA) equipped with a Nucleogel^®^ Ion 300 OA column (Macherey–Nagel, Düren, Germany). Shodex RI101 refractive index detector (Resonac, formerly Showa Denko, Tokyo, Japan).

The reagents and materials were successfully employed in our lab, but similar reagents of different brands could be likely used in most cases. Where a specific material is required, it will be explicitly indicated.

## 3. Procedure

### 3.1. Vector Selection

Several strategies can be employed to generate a plasmid for the overexpression of a gene of interest (GOI) selected from the Tks genome [19]. The first and most critical step is to select a suitable expression vector. Generally, commercially available vectors offer standardised backbones and selectable markers, ensuring compatibility with a wide range of hosts. These vectors can be further customised by modifying the multiple cloning site (MCS), or by inserting an artificial MCS if one is not already present, to facilitate the cloning of the GOI.

#### pBI121-MCS Generation

**Figure 2:**
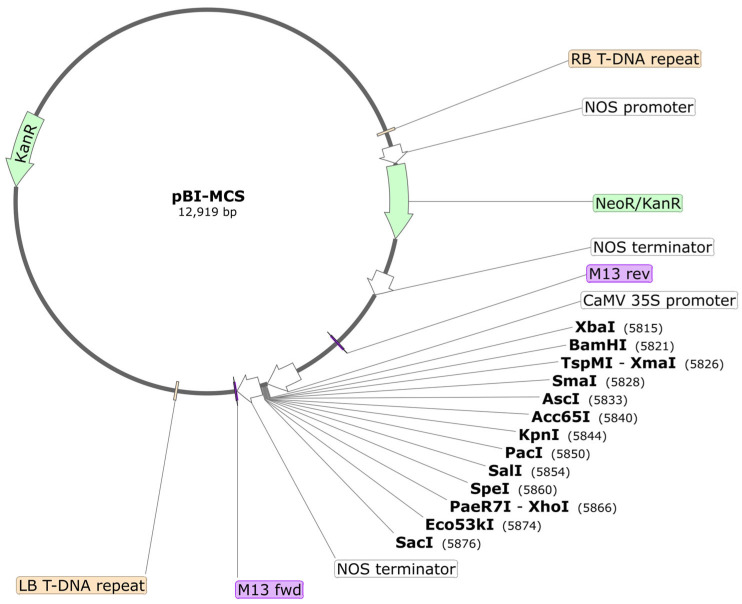
pBI121-MCS map created with SnapGene software v.8.2.2, (www.snapgene.com). The plasmid is represented as a circle. The T-DNA portion of the plasmid starts from the right border (RB T-DNA repeat) and it ends at the left border (LB T-DNA repeat). Between LB and RB the T-DNA contains the NeoR/KanR gene (green arrow) under the control of NOS promoter (white arrow) and terminator (white arrow), wich confers kanamycin resistance in transformed plants. Between the CaMV 35S promoter (white arrow) and an additional NOS terminator (white arrow) the multicloning site is evidenced with the restriction enzyme names reported in bold and their position reported between parenthesys. The position of M13 fwd and rev primers is highlighted in violet. Outside the T-DNA region the position of the kanamycin resistance gene in bacterium (KanR) is shown as a green arrow. The map file, containing a description of all the features, was included in the Supplementary Materials, and it can be visualised and explored with the Snapgene viewer software freely available at www.snapgene.com/snapgene-viewer (websites accessed on 23 January 2026).

This section reports the steps for constructing pBI121-MCS, a modified version of the Agrobacterium binary vector pBI121 [20,21], which contains a multiple cloning site (MCS) in place of the GUS gene under the control of the constitutive CaMV 35S promoter (Figure 2).

We used the pBI121 binary vector due to its proven stability, broad compatibility with *Agrobacterium tumefaciens*, and excellent performance in the previous literature [14]. These features make it a reliable choice for generating transgenic lines and functional studies in this species.

The original pBI121 vector harbours only XbaI, BamHI, XmaI/SmaI between the CaMV 35S promoter and GUS, and SacI between GUS and the nos terminator.

Removing GUS allows direct insertion of any gene of interest in its place, enabling the production of recombinant constructs without interference from this reporter gene. This modification increases the flexibility of the vector for functional studies, overexpression experiments, and other molecular applications where the presence of GUS is unnecessary or would complicate downstream analyses. The introduction of additional restriction sites facilitates the cloning of a gene of interest (GOI). The T-DNA region of the plasmid contains the nptII gene, which provides resistance to kanamycin, and is under the control of the constitutive nos promoter.

The steps required for plasmid modification are as follows:

- Generate the insert containing the modified MCS by performing high-fidelity PCR with Q5 polymerase, using pBI121 as the template. Use primers 35S-for (5′-CTATCCTTCGCAAGACCCTTC-3′) and GUS-MCS-rev (5′-GGTGGTGAGCTCCTCGAGACTAGTGTCGACTTAATTAAGGTACGGCGCGCCGGTAGCAATTCCCGAGGCT-3′) to amplify a fragment of 2020 bp containing the GUS gene, and to introduce the MCS sequence at the reverse end.
- After amplification, check the PCR product on agarose gel and purify it with the MinElute PCR Purification Kit.
- Digest both the purified PCR product and pBI121 backbone with XbaI and SacI. 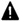 **CRITICAL STEP:** Include 0.1 units/µL of rSAP in the vector-restriction reaction to prevent self-ligation. Phosphatase removes the 5′-phosphate group from the linearised vector ends, which are required for ligation by DNA ligase. By dephosphorylating the vector, the probability of the vector re-circularising without an insert is greatly reduced, thereby increasing the efficiency of cloning the desired fragment.
- Heat-inactivate the digestion reactions at 65 °C for 20′. Purify them by column purification to obtain clean substrates for ligation.
- Combine insert and vector at an appropriate molar ratio, typically using a two- to five-fold excess of insert over vector. Perform the ligation with T4 DNA ligase under standard conditions.
- Use standard procedures to transform the ligation mixture into competent *E. coli* cells and plate the cells on selective medium containing kanamycin.
- Screen the resulting colonies by PCR with primers 35S-for and nos-rev (5′-CAATTATACATTTAATACGCG-3′) to confirm the presence of the correct insert. Grow positive colonies overnight in liquid LB.
- Isolate plasmid DNA using the Plasmid Miniprep Kit. Verify the construct via restriction analysis and Sanger sequencing.
- Digest the resulting plasmid (pBI121-GUS-MCS) with SmaI to remove the GUS sequence. The GUS gene is unnecessary for the overexpression experiment, and we suggest removing it to obtain a simpler vector with just a general-purpose MCS between promoter (35S) and nos terminator sequences. Separate the linearized plasmid backbone from the excised fragment via agarose gel electrophoresis and recover the backbone by gel extraction.
- Re-ligate the purified backbone to generate the final construct pBI121-MCS carrying the new MCS (XbaI, BamHI, XmaI/SmaI, AscI, KpnI, PacI, SalI, SpeI, XhoI, SacI) between the CaMV 35S promoter and the nos terminator. Transform the ligation mixture into competent *E. coli* cells and screen colonies by PCR or restriction digestion.
- Sequence plasmids from positive colonies across the junctions to confirm correct insertion and orientation of the MCS, using primers 35S-for and nos-rev.

### 3.2. Overexpression Vector Construction

In this section, the steps necessary to generate a plasmid for the overexpression of a GOI selected from the Tks genome are shown.

PCR primers have to be designed to amplify the whole CDS (Coding DNA Sequence) of the GOI, selected from the available Tks genome sequence [19] or a similar source. From 5′ to 3′, the forward primer sequence must include a leader motif (e.g., GGTGGT) followed by a first restriction site sequence (RS1, e.g., BamHI) and the first 18–22 bases of the CDS, including the start codon. Similarly, from 5′ to 3′, the reverse primer sequence must include a leader motif (e.g., GGTGGT) followed by a second restriction site sequence (RS2, e.g., SacI) and the reverse complement of the last 18–22 bases of the CDS, including the stop codon. The presence of short leader sequences added to the 5′ end of the primers improves the efficiency of restriction enzyme digestion. Many restriction enzymes require a few extra nucleotides to flank their recognition site in order to bind to and cleave DNA efficiently. The leader motif (e.g., GGTGGT) provides the necessary overhang upstream of the restriction site. Specific requirements for leader motifs can be found in restriction enzyme manuals or on manufacturer websites.

Selected restriction sites must not be present within the CDS sequence of the GOI. If none of the restriction sites available in the MCS are absent in the selected CDS, a partial digestion could be performed (described below). Preferentially, use enzymes with compatible buffers to perform a single digestion reaction. In the following text, the primers will be referred to as RS1-start-for and RS2-stop-rev. These primers have the following general sequence:

RS1-start-for: 5′-GGTGGT-RS1-ATG…-3′

RS2-stop-rev: 5′-GGTGGT-RS2-CTA or TCA or TTA…-3′

- Extract total RNA with InnuPrep Plant RNA kit from a tissue or organ where the GOI is expected to be expressed and proceed to first-strand cDNA synthesis using SuperScript™ IV Reverse Transcriptase.
- Amplify the GOI CDS from cDNA via PCR using RS1-start-for and RS2-stop-rev primers and a high-fidelity polymerase such as Q5 High-Fidelity DNA Polymerase according to the manufacturer’s instructions.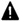 **CRITICAL STEP:** The use of a high-fidelity DNA polymerase is mandatory to avoid random point mutations caused by standard Taq DNA polymerase.
- Verify amplicon size by electrophoresis on 1.5% TAE agarose gel.
- Digest both the vector and the amplicon with the selected pair of restriction enzymes according to the manufacturer’s instructions.
- Purify the digested amplicon using a PCR purification kit such as the Qiagen MinElute PCR Purification Kit.
- Purify the linearised vector using size-exclusion resins such as Sephadex G-50 or Sepharose CL-6B. Do not use a PCR purification column or the linearised plasmid will remain stuck to it. 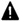 **CRITICAL STEP:** Columns designed for PCR products are optimised for smaller DNA fragments and may retain or damage large plasmid DNA, resulting in low recovery. Size-exclusion chromatography using resins such as Sephadex G-50 or Sepharose CL-6B provides efficient separation and recovery of intact linearised plasmid molecules.
- Ligate the insert and the vector with a molar ratio from 2:1 to 5:1 using T4 DNA Ligase according to the manufacturer’s instructions.
- Transform the ligation product into electrocompetent DH5 alpha *E. coli* cells using a standard electroporation procedure and grow overnight at 37 °C on a LB agar plate containing 100 mg/mL kanamycin.
- Screen at least 10 colonies via colony PCR for the presence of the desired insert with primers 35S-for and RS2-stop-rev and using the empty pBI121-MCS as a negative control. Visualise PCR products after electrophoresis on 1.5% TAE agarose gel and check for the presence of an amplicon of the expected size.
- Inoculate liquid LB medium containing 100 mg/mL kanamycin with a single positive colony and incubate overnight at 37 °C in an orbital shaker at 170 rpm.
- Perform plasmid extraction using the Plasmid Miniprep Kit following the manufacturer’s instructions. Store at −20 °C.
- Confirm the pBI-GOI vector via restriction analysis and sequencing to ensure the correct insertion and sequence of the GOI CDS fragment. Use 35s-for and nos-rev primers for 5′ and 3′ sequencing and, if the GOI CDS is longer than 1 kb, use additional forward and reverse primers internal to the CDS. Once the sequence is confirmed, the plasmid can be used as a binary vector for *Agrobacterium*-mediated plant transformation.

### 3.3. Vector Transformation into Agrobacterium tumefaciens

**Figure 3:**
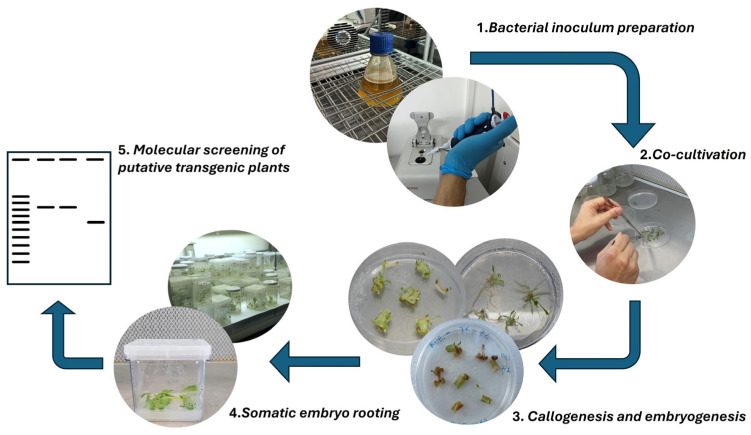
Graphical representation of the main steps of plant transformation starting from Tks leaf explants using *Agrobacterium tumefaciens* strain *EHA105*.

For the transformation of Tks plants, we tested two strains of *Agrobacterium tumefaciens*: EHA105 and AGL1, obtaining higher transformation efficiency with the first strain. The whole procedure is summarised in Figure 3.

- Transform *Agrobacterium tumefaciens* electrocompetent EHA105 cells with the pBI-GOI vector using the MicroPulser Electroporator: mix 1–2 µL of plasmid DNA with 50 µL of electrocompetent EHA105 cells, then transfer to a pre-chilled 0.1 cm cuvette. Use the Agrobacterium-specific setting (2.2 kV voltage without pulse truncation) then immediately add 1 mL of LB medium. Alternatively, any standard electroporation procedure for *Agrobacterium* cells is fine.
- Recovery of *Agrobacterium* cells is performed at 28 °C with shaking for 4 h without antibiotics.
- After recovery, plate on selective LB agar containing 100 mg/L rifampicin and 100 mg/L kanamycin. Incubate at 28 °C until colonies appear (about 48 h).
- Screen resulting colonies by colony PCR as described in Section 3.2, step 10.
- Inoculate 50 mL of LB medium supplemented with 100 mg/L rifampicin and 100 mg/L kanamycin by picking a single positive colony. Incubate at 28 °C with 200 rpm shaking until culture OD_600_ reaches a value of about 0.6 (24 h can be sufficient).
- Prepare several glycerol stocks from the culture by mixing 500 µL of culture with 500 µL of sterile glycerol and freezing in liquid nitrogen. Store at −80 °C. Use stocks as starters to inoculate the cultures that will be employed for plant transformation.

### 3.4. Agrobacterium tumefaciens-Mediated Plant Transformation

#### 3.4.1. Preparation of Plant Material (1 Week)

- Under a laminar flow hood, sterilise Tks seeds by rinsing 3′ in 25% commercial bleach in bdH_2_O (bidistilled water).
- Rapidly wash seeds 3 times in bdH_2_O.
- Place sterile seeds in Petri plates (20 seeds/plate) containing 20 mL solid bdH_2_O (autoclaved bdH_2_O with 10% agar). Put in a growth chamber at 23 °C with a 16/8 photoperiod until germination.
- Transfer germinated seedlings in vivo to 2.6 L polyethylene pots containing a commercial substrate consisting of decomposed peat moss supplemented with 7% horticultural sand (*v*/*v*) to improve drainage. Peat moss, which is partially decomposed sphagnum moss, is a common ingredient in horticultural substrates. It is highly porous, with good water- and nutrient-holding capacity. This provides a stable, aerated medium for root development. In this protocol, the addition of 7% horticultural sand to the peat moss improves drainage and prevents waterlogging, ensuring optimal growth conditions for the transferred seedlings. Alternatively, seedlings can be transferred in magenta boxes with 40 mL MS1/2B5 without hormones and maintained in vitro by monthly transfer to fresh medium. Magenta boxes are commercially available, autoclavable plastic culture vessels commonly used for plant tissue culture. They provide a sterile, contained environment suitable for in vitro growth and maintenance of seedlings and small plants. As an alternative, any transparent vessel that can be properly sealed and autoclaved can be used.

**OPTIONAL STEP.** Tks is a sexually reproducing allogamous species; thus, existing accessions are characterised by a certain degree of genetic and phenotypic variability. Available accessions include segregating the population propagated by seed. In Tks, we observed a high degree of variability in the ability to produce callus and somatic embryos among individual plants. To better standardise the experimental conditions, we suggest performing a preliminary selection of plants with higher regeneration ability. The regeneration steps described below can be applied to preliminarily select a plant with higher regeneration ability. In our lab, we used a plant from the high-rubber accession W6 35166 [1], named Tks-G. This plant was maintained in vitro and clonally propagated as described in Section 3.7.

#### 3.4.2. Bacterial Inoculum Preparation (2 Days)

- Inoculate a sterile flask containing 50 mL of LB liquid culture medium with 100 mg/L kanamycin and 100 mg/L rifampicin with a glycerol stock obtained as described in Section 3.3.
- Incubate at 28 °C for 24 h under shaking conditions.
- Assess the bacterial growth using a spectrophotometer. When the OD600 is between 0.4 and 0.8, recover the pellet by centrifuging at 4000 rcf for 30 min.
- Resuspend pellet in 20 mL sterile distilled water.

#### 3.4.3. Infection and Co-Cultivation (2–3 Days)

The starting material for the infection can be collected from non-sterile ex vivo plants or sterile in vitro plants.

If the starting material is from non-sterile ex vitro plants, the explants need to be sterilised as described in the procedure below.

- Sample young Tks leaves from the inner part of the rosette with a sterile blade and forceps and place in sterile bdH_2_O. All subsequent steps are performed under a laminar flow hood in sterility conditions.
- Quickly rinse the sampled leaves in 70% ethanol and immediately wash three times in sterile bdH_2_O.
- Rinse in a solution of 25% commercial bleach supplemented with one drop of Tween-20 for 5 min with occasional inversion. Then, wash leaf explants three times with sterile bdH_2_O and leave in sterile water until required.

If the starting material is from an in vitro sterile plant, sterilisation is unnecessary.

- Sample young Tks leaves from the inner part of the rosette with a sterile blade and forceps and place in sterile bdH_2_O under a laminar flow hood in sterility conditions.

From here on, the procedure is the same for both ex vitro and in vitro starting materials.

- Prepare explants from sterilised leaves in a Petri plate containing sterile water to avoid excessive desiccation by cutting transversally into pieces of about 0.5 cm, using a sterile scalpel.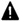 **CRITICAL STEP:** Keep 10–20 control explants separate to avoid contamination with *Agrobacterium*. Control explants, in the last step of the procedure described in this section, will be split into two groups: one with antibiotics (selection control) and one without antibiotics (positive embryogenesis control). The first group, consisting of non-transformed explants exposed to the selective agent, is included to confirm the efficacy of selection and to ensure that only transformed tissues survive and regenerate. What is normally observed is that explants in this group become necrotic and die. The second group provides a baseline for normal growth and development. What is normally observed in this group is that explants quickly produce calli and embryos.
- For the infection phase transfer, cut explants in a Petri dish containing the *Agrobacterium* suspension and incubate at room temperature for 30–60 min.
- Transfer the explants to Petri plates containing solid MSB5 medium. Use 10–15 explants/plate. Use 2 plates for untreated control explants. Seal with Parafilm.
- Place in growth chamber at 23 °C with a 16/8 photoperiod.
- In around two days, *Agrobacterium* becomes visible close to the leaf explants. 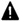 **CRITICAL STEP:** Do not let *Agrobacterium* overgrow on explants.
- Decontaminate explants by washing three times in sterile bdH2O with 400 mg/L Augmentin added under a laminar flow hood. EHA105 cells are susceptible to Augmentin; thus, the use of this antibiotic here and in later steps is necessary to eliminate residual *Agrobacterium* contamination.
- Tap explants on sterile filter paper and transfer to fresh plates containing MSB5 medium supplemented with kanamycin (25 mg/L) and Augmentin (200 mg/L) to select the transformed cells.
- Perform the same treatment to control explants and transfer to two fresh MSB5 plates, one with antibiotics as indicated above (selection control) and one without antibiotics (positive embryogenesis control).

#### 3.4.4. Callogenesis and Embryogenesis (3–4 Weeks Before First Embryos Appear)

- Incubate the plates in the growth chamber at 23 °C with a 16/8 photoperiod.
- Transfer the explants to fresh medium supplemented with antibiotics every 2–3 weeks until callus formation. Augmentin can be reduced after first transfers if no bacterial growth is observed. After about 10–15 days, calli begin to form mainly from the cut close to the central leaf vein.
- As soon as calli grow and begin to form embryos, transfer to separate Petri dishes and label each callus with a univocal numeric identifier. Later, plants deriving from different calli will be considered as originating from independent transformation events.

#### 3.4.5. Rooting

- Separate plantlets from calli when they appear as few-millimetre, unrooted rosettes of green leaves and transfer them to MS1/2B5 rooting medium. Add Augmentin only in case of *Agrobacterium* growth.
- Transfer every 2–3 weeks to fresh medium until roots form. Some plantlets develop roots quickly, while others require more time/transfers.
- Transfer rooted plantlets to magenta boxes containing MS1/2B5 medium without hormones or antibiotics for further development before in vivo transfer.
- Keep the magenta boxes in the growth chamber at 23 °C with a 16/8 photoperiod. If necessary, transfer to fresh medium monthly.

### 3.5. Molecular Screening of Putative Transgenic Plants

- In this phase, rooted plantlets can be examined for the presence of the transgene via PCR. Extract the genomic DNA from one leaflet using the InnuPrep Plant DNA Kit following the manufacturer’s protocol. Spectrophotometrically evaluate DNA quantity and quality.
- Perform the PCR amplification on 50–100 ng of DNA using 35S-for primer and a GOI-specific reverse primer. Use plasmid DNA as a positive control and DNA from wild-type plants as a negative control.
- Evaluate the presence of the specific amplicon by electrophoresis on a 1.5% TAE agarose gel.
- Positive transformed plants can be further analysed for GOI gene expression relative to the wild-type plants via RT-qPCR using gene-specific primers.

### 3.6. Ex Vitro Transfer (2–4 Weeks)

**Figure 4:**
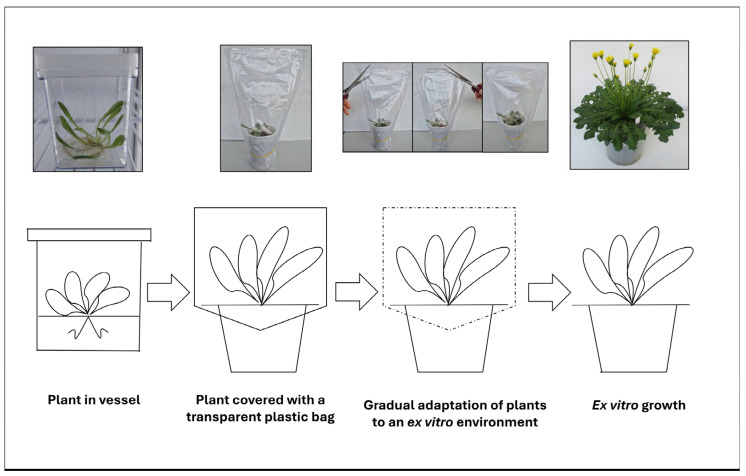
Procedure for the transfer of ex vitro plants. Progressive acclimatisation of in vitro Tks plant is achieved by covering with a plastic bag after transplantation and subsequent gradual bag opening.

Once transgenic Tks plantlets show a well-developed root apparatus, they can be acclimatised gradually to non-sterile ex vitro growth conditions. The following steps describe the procedure for transferring fully developed plants from in vitro culture to soil, enabling them to establish proper roots and adapt to the external environment (Figure 4).

- Once plants are fully developed and a good radical apparatus is formed, carefully extract plants from magenta boxes avoiding damages to radical apparatus. Clean roots from excess agarised substrate residues.
- Place in 100 mL pots containing 50% sand and 50% cultural substrate, as described in Section 3.4.1. A high amount of sand can reduce possible root rot in this phase. Water and cover with a transparent plastic bag. The transparent plastic bag is used to maintain a humid environment around the seedlings during the initial transition from in vitro to ex vitro conditions. Plants grown in vessels develop in high-humidity environment and have a limited ability to regulate transpiration. Covering the pots with a transparent plastic bag enables gradual acclimatisation by reducing water loss and preventing desiccation, thus facilitating the transition from closed, sterile-culture conditions to non-sterile open-pot cultivation. Transfer to a growth chamber dedicated to non-sterile plant cultivation.
- After about a week, once new leaves begin to form, start cutting the corners of the plastic bag in order to gradually adapt the plants to the external environment.
- Completely remove the bag after about one more week. At this stage, the plants can grow for about one month in 100 mL pots and later can be transferred to larger (i.e., 2.6 L) pots for further development.

### 3.7. In Vitro Plant Propagation via Root Cuttings

**Figure 5:**
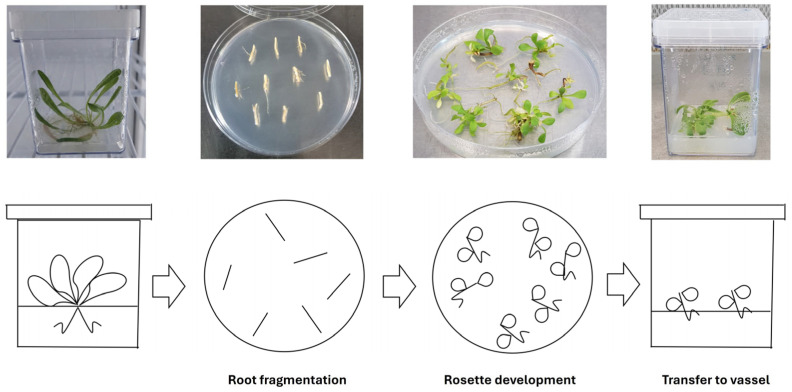
Procedure to propagate plants via root cuttings in vitro.

This section describes a rapid approach for cloning Tks plants in vitro from root cuttings to obtain a population of genetically identical plants ready for subsequent experiments (Figure 5).

- Under a laminar flow hood, take root portions from well-rooted Tks plants grown in magenta boxes and cut into small pieces measuring 1–2 cm.
- Place 10–15 root cuttings on Petri plates containing MS½B5 culture medium without hormones.
- Incubate the plates in the growth chamber at 23 °C with a 16/8 photoperiod. After about 3 weeks, rosettes will develop from root cuttings.
- Once new roots begin to develop from rosettes, transfer each plantlet to magenta boxes containing MS½B5 culture medium without hormones. Up to four plantlets can be grown in each box. Once full development is reached, transfer to ex vitro conditions as described.

### 3.8. In Vivo Tks Plant Propagation by Root Cuttings

**Figure 6:**
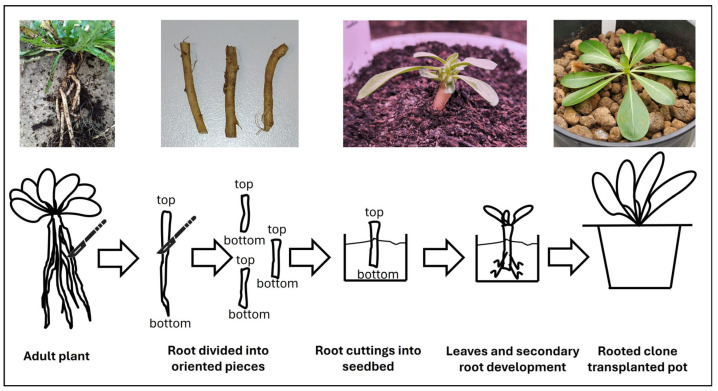
Procedure to propagate plants via root cuttings in vivo.

Plants in vivo can be easily propagated via root cuttings using the procedure described below (Figure 6). Here, the limiting factor is the availability of a good root apparatus from which a sufficient number of good quality cuttings can be obtained.

- Prepare growth substrate by mixing standard commercial culture substrate (decomposed peat moss) with 7% horticultural sand (*v*/*v*) to improve drainage. Put substrate in a plastic seed tray with diameter holes of about 5 cm.
- With a sterile scalpel, prepare the Tks plants root cuttings (about 4 cm long) from a well-developed root (more than 0.4 mm diameter).
- Plant cuttings by dipping 2/3 of their length in the right orientation on the prepared substrate, with two to three explants per seed tray hole. 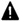 **CRITICAL STEP:** Ensure that the cuttings are placed in the correct apical–basal orientation, as inversion can severely reduce rooting and regeneration efficiency.
- Cultivate plants in growth chamber at 23 °C with a 16/8 photoperiod. A rosette begins to develop from each cutting after around two weeks.
- Transplant the Tks cuttings into 2.6 litre polyethylene pots once they have fully developed into plantlets. This step requires at least one month.

### 3.9. Natural Rubber, Inulin, and Resin Quantification in Tks Plants

Accurate quantification of NR content is essential for advancing the development of alternative rubber crops and ensuring their competitiveness within the natural rubber industry. Over the years, various analytical techniques have been employed to quantify rubber content, including gravimetric analysis [22], gel permeation chromatography [23], infrared spectroscopy [24], and mass spectrometry [25], among others. In this section, we describe two complementary gravimetric methods for phenotyping Tks plants based on their rubber content. Gravimetry is a classical approach that does not require sophisticated analytical instrumentation; thus, it is cost-effective when compared to the time-consuming and expensive above-mentioned analytic techniques; nevertheless, it demands highly calibrated and sensitive weighing conditions.

**Figure 7:**
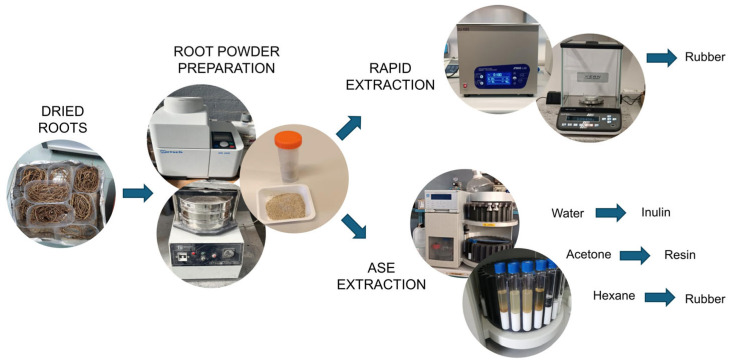
Schematic representation of phenotyping procedure with rapid and ASE (Accelerated Solvent Extraction) extraction methods.

The first protocol (Section 3.9.2) is a rapid screening method, suitable for processing many plants in a short time. Although it is less precise, it enables the efficient selection of promising individuals for further analysis. The second protocol, described in Section 3.9.3, is based on Accelerated Solvent Extraction (ASE) modified from [6]. It allows the accurate quantification of the three major classes of compounds in Tks roots: natural rubber, inulin (a fructan polysaccharide), and resins (a mixture of soluble, non-rubber, and organic extractives). This approach allows the simultaneous determination of all three components from the same root sample, making it particularly valuable for genotype comparisons. Moreover, it provides a comprehensive chemical profile of the root material, which is particularly useful for characterising candidate genotypes identified through the rapid screening method. The whole procedure is summarised in Figure 7.

#### 3.9.1. Preparation of Dried Root Powder Samples (2–3 Days)

- Collect root pools from at least five plants of each genotype. Consider at least three replicates for each determination.
- Wash the roots thoroughly with tap water.
- Dry the roots in an oven at 45 °C until they reach a constant weight.
- Cut dried roots into ~1 cm long pieces.
- Grind these pieces in an ultracentrifugal mill and pass the resulting powder through a 3 mm sieve. Proceed with Section 3.9.2 or Section 3.9.3.

#### 3.9.2. Rapid Extraction and Quantification of Rubber (2 Days)

- Weigh 100 mg to 1 gr of root powder and add 20 mL of acetone per gram of powder.
- Perform three ultrasonic treatment cycles (6 min for each cycle) for the acetone suspension using an ultrasonic cleaning bath at 80% power and a frequency of 40 kHz.
- After each cycle, centrifuge at 3500 rpm for 10 min and discard the supernatant.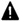 **CRITICAL STEP:** This step removes resins from the samples.
- Repeat the above ultrasonic treatment procedure (step b), replacing acetone with hexane (ratio 1:20, *w*/*v*).
- Collect hexane fractions after centrifugation and transfer them to pre-weighed aluminium weighing boats.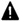 **CRITICAL STEP:** Aluminium weighing boats need to be conditioned before using them. To this end, incubate them at 50 °C overnight or until constant weight.
- Allow hexane to evaporate under a fume hood until constant weight is achieved and determine rubber yield via gravimetric analysis of the dried extract, expressed relative to the dry weight of the roots.

#### 3.9.3. ASE and Quantification of Rubber (3–4 Days)

- Place a cellulose filter at the bottom of an 11 mL extraction cell and fill the cell with 20–30 mesh inert material.
- Add 0.5 g of ground roots (Section 3.9.1), mixing continuously with a metal spatula before sealing the cell.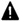 **CRITICAL STEP:** Mix the powder gently and continuously as you add it to prevent channelling and to achieve uniform packing. Avoid compacting the sample too tightly and keep the bed lightly packed to facilitate uniform solvent flow and ensure the reproducibility of extraction processes.
- Set up the ASE instrument to perform the extraction cycles as follows:
  ○ Water extraction: two cycles of 40 min each at 95 °C.
  ○ Acetone extraction: one cycle of 40 min at 40 °C.
  ○ Hexane extraction: three cycles of 20 min each at 160 °C.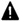 **CRITICAL STEP:** Use the ASE’s normal rinse and purge cycles between solvent changes as recommended by the instrument. Verify that the ASE solvent lines and seals are clean and that the temperature and pressure settings match the solvent boiling point and instrument recommendations.
- Collect the water, acetone, and hexane fractions after each extraction step.
- Transfer the acetone and hexane fractions into pre-weighed aluminium weighing boats. Evaporate the solvents under a fume hood until a constant weight is reached.
- Determine the resin and rubber yields by performing a gravimetric analysis of the acetone- and hexane-dried extracts, respectively.
- Use water extract for inulin quantification via high-performance liquid chromatography (HPLC), as described in Section 3.9.4.

#### 3.9.4. Inulin Extraction, Hydrolysis, and Quantification (1 Day)

**OPTIONAL STEP:** Centrifuge the ASE water fraction at 3500 rpm for 5 min to remove any residual solids. Collect 2 mL of the clear supernatant for subsequent analysis.

- Add 4 mL of 0.75 M HCl to 2 mL of the ASE water fraction and transfer the acidified solution containing inulin to a heat-shake system.
- Hydrolyse it for 15′ at 100 °C with agitation at 500 rpm.
- Centrifuge the hydrolysed solution at 3500 rpm for 5 min and filter the supernatant through a 0.45 µm PTFE Whatman® filter.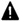 **CRITICAL STEP:** Ensure complete filtration of the sample through a 0.45 µm PTFE filter. Any remaining particulates may damage the HPLC column or cause unstable baseline signals. Use clean, new filters and avoid applying excessive pressure during filtration.
- Analyse carbohydrates as described in [26], using an HPLC DX 300 system equipped with a Nucleogel^®^ Ion 300 OA column. Use 10 mN H_2_SO_4_ as the mobile phase and detect the compounds with a Shodex RI101 refractive index detector.
- Calculate the inulin content (%) according to the following equation:

Inulin % = (Cf + Cg) × 0.9 × 3 CR × 100

where Cf and Cg are the concentration in g/L of fructose and glucose, respectively; 0.9 is the correction factor applied for the oligomer-to-monomer hydration; 3 is the dilution factor for the HCl hydrolysis; CR is the concentration in g/L of the initial suspended roots.

## 4. Reagent Setup

In this section, we report the composition of media used in *Agrobacterium tumefaciens*-mediated plant transformation: MSB5 (Table 1); MS1/2B5 (Table 2). Preparation of hormones and antibiotics stock solutions is also described. The main difference between MSB5 and MS1/2B5 is that both MS salt and sucrose are reduced to half the amount. MSB5 is used from the initial steps until development of plantlets. Subsequently, green plantlets are separated from the callus and put on half-strength medium to promote heterotrophic carbohydrate metabolism and to induce further development and rooting. IAA and Zeatin are added to MSB5 as this combination of hormones promotes callogenesis. NAA is added to MS1/2B5 plates to induce rooting. NAA can be omitted once plantlets develop roots.

Both IAA and NAA are synthetic auxins commonly used in in vitro culture experiments. Auxins are necessary both in the step of callus induction and in the rooting step.

While IAA was effective in combination with Zeatin for callus induction, we also tested it for rooting, but it was not effective. NAA, at the suggested concentration, was highly effective in inducing rooting in plantlets.

Autoclave the medium with salts, vitamins, sucrose, and agar at 121 °C. After sterilisation and cooling of the medium at 60–65 °C, add hormones and antibiotics from filter-sterilised stock solutions under a laminar flow hood. Transfer about 15–20 mL of the still-liquid medium into Petri dishes and store at 4 °C for up to one month. Prepare hormones and antibiotics stock solution as follows.

- 100× Indole-3-acetic acid (IAA): Dissolve 0.1 g/L of IAA in NH_3_ and dilute with sterile distilled water to volume. The preparation requires the use of a chemical hood. Sterilise by filtration (0.22 µm). Store at 4 °C.
- 100× Zeatin: Dissolve 0.1 g/L of Zeatin in HCl and dilute with sterile distilled water to volume. Store at 4 °C.
- 50× Kanamycin: Dissolve 50 mg of kanamycin in 1 mL of sterile distilled water. Sterilise by filtration (0.22 µm). Store at −20 °C.
- 50× Augmentin: Dissolve 50 mg of Augmentin in 1 mL of sterile distilled. Sterilise by filtration (0.22 µm). Store at −20 °C.

Autoclave the medium with salts, vitamins, sucrose, and agar at 121 °C. After sterilisation and rapid cooling of the medium, add the hormones and antibiotics. Transfer about 15–20 mL of the still-liquid medium into Petri dishes and store at 4 °C.

Prepare the NAA stock solution as follows.

- 100× 1-naphthaleneacetic Acid (NAA): Dissolve 0.1 g/L of NAA in NH_3_ and dilute with sterile distilled water to volume. The preparation requires the use of a chemical hood. Store at 4 °C.

## Figures and Tables

**Table 1:** Composition of MSB5 medium.

Component	Amount for 1 L
MS salt	4.3 g
B5 vitamins [27]	1 mg
Indole-3-acetic acid (IAA)	0.2 mg
Zeatin	1.5 mg
Sucrose	30 g
Agar	8 g
To select transformed plant explants	
Kanamycin	25 mg
Augmentin	200–300 mg
Adjust pH to 5.8	

**Table 2:** Composition of MS1/2B5 medium.

Component	Amount for 1 L
MS salt	2.15 g
B5 vitamins [27]	1 mg
1-naphthaleneacetic Acid (NAA)	0.2 mg
Kanamycin	10 mg
Augmentin	100–200 mg
Sucrose	15 g
Agar	8 g
Adjust pH to 5.8	

## Data Availability

The original contributions presented in this study are included in the article/Supplementary Materials. Further inquiries can be directed to the corresponding author.

## Supplementary Materials

The following supporting information can be downloaded at: www.snapgene.com/snapgene-viewer (websites accessed on 23 January 2026).
